# The Arabidopsis non‐host defence‐associated coumarin scopoletin protects soybean from Asian soybean rust

**DOI:** 10.1111/tpj.14426

**Published:** 2019-07-01

**Authors:** Sebastian F. Beyer, Alexander Beesley, Philipp F.W. Rohmann, Holger Schultheiss, Uwe Conrath, Caspar J.G. Langenbach

**Affiliations:** ^1^ Department of Plant Physiology RWTH Aachen University Aachen 52074 Germany; ^2^ Agricultural Center BASF Plant Science Company GmbH Limburgerhof 67117 Germany

**Keywords:** scopoletin, *Phakopsora pachyrhizi*, *Glycine max*, *Arabidopsis thaliana*, plant protection

## Abstract

The fungus *Phakopsora pachyrhizi* (*Pp*) causes Asian soybean rust (SBR) disease which provokes tremendous losses in global soybean production. *Pp* is mainly controlled with synthetic fungicides to which the fungus swiftly develops fungicide resistance. To substitute or complement synthetic fungicides in Asian soybean rust control, we aimed to identify antifungal metabolites in Arabidopsis which is not a host for *Pp*. Comparative transcriptional and metabolic profiling of the *Pp*‐inoculated Arabidopsis non‐host and the soybean host revealed induction of phenylpropanoid metabolism‐associated genes in both species but activation of scopoletin biosynthesis only in the resistant non‐host. Scopoletin is a coumarin and an antioxidant. *In vitro* experiments disclosed fungistatic activity of scopoletin against *Pp*, associated with reduced accumulation of reactive oxygen species (ROS) in fungal pre‐infection structures. Non‐antioxidant and antioxidant molecules including coumarins with a similar structure to scopoletin were inactive or much less effective at inhibiting fungal accumulation of ROS and germination of *Pp* spores. When sprayed onto Arabidopsis leaves, scopoletin also suppressed the formation of *Pp* pre‐infection structures and penetration of the plant. However, scopoletin neither directly activated defence nor did it prime Arabidopsis for enhanced defence, therefore emphasizing fungistatic activity as the exclusive mode of action of scopoletin against *Pp*. Because scopletin also protected soybean from *Pp* infection, the coumarin may serve as a natural fungicide or as a lead for the development of near‐to‐nature fungicides against Asian soybean rust.

## Significance Statement

Plants with non‐host resistance to a given pathogen harbour promising traits for effective plant protection. We report that upon inoculation with the Asian soybean rust‐causing fungus *Phakopsora pachyrhizi* the coumarin scopoletin specifically accumulates in the Arabidopsis non‐host, but not the soybean host. Because exogenously applied scopoletin scavenges ROS in the pre‐infection structures of *P. pachyrhizi*, inhibits spore germination, and protects soybean from Asian soybean rust the coumarin may qualify as a natural fungicide.

## Introduction

Soybean is the most important legume crop with a global production volume of approximately 335 million tonnes in 2016 (FAOSTAT, 2018; http://www.soystats.com). Due to its high content of proteins, oil and essential amino acids soybean is crucial for satisfying the demands of a growing world population for food and feed (Lusas, 2004). However, Asian soybean rust (SBR), a devastating disease caused by *Phakopsora pachyrhizi* (*Pp*) poses a major threat to soybean production and world food security (Pennisi, 2010). No commercial soybean variety is resistant to all isolates of *Pp* (Goellner *et al*., 2010; Langenbach *et al*., 2016a) and single gene‐mediated resistance is quickly overcome due to the vast genetic variability of the pathogen (Yorinori *et al*., 2005; Garcia *et al*., 2008; Yamaoka, 2014). Therefore, SBR is currently mainly controlled with synthetic fungicides and their various mixtures, primarily those in the strobilurin (Quinone‐outside‐inhibitors; QoIs), triazole (demethylation inhibitors, DMIs), and succinate dehydrogenase inhibitor (SDHI) classes (Godoy *et al*., 2015, 2016; Langenbach *et al*., 2016a). However, extensive fungicide use enhances the selection pressure on the pathogen and therefore promotes the evolution of fungicide‐resistant strains (Godoy, 2012). For example, some field isolates of *Pp* carry mutations in *CYP51* (encoding sterol 14α‐demethylase) or *CYTB* (encoding cytochrome b) and are less sensitive to QoI and DMI fungicides (Schmitz *et al*., 2014; FRAC, 2015; Klosowski *et al*., 2016). Recently, a *Pp* variant with reduced sensitivity to SDHIs has been discovered in Brazil (Simões *et al*., 2018) only a few years after the first application of commercial SDHI‐containing fungicide mixtures in this country (Godoy *et al*., 2016). Therefore, even old multisite contact fungicides such as mancozeb or copper sulfate are currently being reconsidered for use in SBR control (Godoy *et al*., 2016). In addition to promoting the selection of fungicide‐insensitive strains, the use of some chemical fungicides raises ecological and health concerns (Ochoa‐Acuña *et al*., 2009; Pearson *et al*., 2016). Therefore, non‐hazardous but effective compounds with inhibitory activity to *Pp* may complement or even replace existing SBR management strategies and promote sustainable control of SBR.

Natural plant‐protection compounds and biocontrol measures are not yet implemented in current SBR management practices. However, they may help constraining SBR in the future. For example, natural inducers of disease resistance in soybean (e.g. beneficial microbes, plant volatiles), mycoparasites, and plant oils are reported to suppress fungal development or sporulation (reviewed by Langenbach *et al*., 2016a). Silicon and phosphite have both been reported to act dually by stimulating plant defence and directly inhibiting *Pp*, either by antagonizing fungal growth or building up a penetration barrier in the soybean cuticle (Ma and Yamaji, 2006; da Cruz *et al*., 2013; Gill *et al*., 2018). Secondary metabolites in SBR‐resisting non‐host plants may also represent promising tools for eco‐friendly SBR control. For example, the isoflavonoid phytoalexins medicarpin and ononin antagonize development of *Pp* pre‐infection structures (Ishiga *et al*., 2015) and may therefore be employed as natural fungicides.

The phenylpropanoid compound 7‐hydroxy‐6‐methoxycoumarin (scopoletin) occurs in many plants (Gnonlonfin *et al*., 2012). Scopoletin has antimicrobial, viricidal and insecticidal activity (Tal and Robeson, 1986a; Goy *et al*., 1993; Prats *et al*., 2002, 2006; Gómez‐Vásquez *et al*., 2004; Adfa *et al*., 2010; Tripathi *et al*., 2011; Gnonlonfin *et al*., 2012; Stringlis *et al*., 2018). In the Arabidopsis shoot, scopoletin accumulates after microbial attack and upon treatment with 2,4‐dichlorophenoxyacetic acid. In the Arabidopsis root, scopoletin is constitutively present and further induced and secreted into the rhizosphere during iron deficiency (Kai *et al*., 2008; Fourcroy *et al*., 2014; Schmid *et al*., 2014; Schmidt *et al*., 2014; Tsai and Schmidt, 2017). Stringlis *et al*. (2018) recently disclosed that scopoletin exudates can shape the root microbiome by selectively suppressing soil‐borne fungal pathogens while promoting plant‐beneficial rhizobacteria that trigger the induced systemic disease resistance response. In both the Arabidopsis root and shoot, scopoletin accumulation tightly correlates with the expression of the *FERULOYL‐CoA 6‐HYDROXYLASE1* (*F6′H1*) gene, which encodes the key enzyme of scopoletin biosynthesis in this plant (Kai *et al*., 2008; Schmid *et al*., 2014). Due to its specificity in inhibiting pathogens rather than beneficial organisms (Stringlis *et al*., 2018) the coumarin has huge potential for use as a natural plant‐protection compound. In addition its structure may serve a lead for the synthesis of near‐natural fungicides to fight SBR.

Using whole‐genome expression profiling we previously characterized a set of *POSTINVASION‐INDUCED NHR GENEs* (*PING*s) whose expression is specifically linked to post‐invasion non‐host resistance (NHR) of Arabidopsis to *Pp* (Langenbach *et al*., 2016b). *PINGs* were exclusively activated in the *Pp*‐inoculated Arabidopsis *penetration2* (*pen2*) mutant with impaired pre‐but intact post‐invasion NHR. Their expression was absent in both penetration resistant Arabidopsis wild‐type and the *pen2 pad4 sag101* (for *pen2*/*phytoalexin‐deficient 4*/*senescence‐associated gene 101*) mutant with impaired pre‐ and post‐invasion NHR to *Pp* (Langenbach *et al*., 2013, 2016b). Transfer of various *PINGs* from the Arabidopsis non‐host to the soybean host reduced the susceptibility of the soybean cultivar to SBR (Langenbach *et al*., 2016b). This finding demonstrates that Arabidopsis harbours useful traits for soybean protection from SBR. Here, we aimed at identifying NHR‐associated secondary metabolites in Arabidopsis to investigate their potential as natural fungicides to fight SBR. We reasoned that SBR‐susceptible soybean plants apparently do not produce antifungal metabolites in sufficient amounts and/or at the required time to effectively defeat the fungus. However, non‐host plants, such as Arabidopsis, would be a valuable source of metabolites that would effectively inhibit *Pp*. To identify such metabolites, we first compared the transcriptional activation of genes in the phenylpropanoid pathway (PPP) and metabolic changes in leaves of both the Arabidopsis non‐host and soybean host after challenge with *Pp*. As scopoletin biosynthesis was induced in Arabidopsis but not soybean, we evaluated the capacity of scopoletin to protect soybean from SBR. Here, we show that foliar application of scopoletin provides SBR protection by directly inhibiting the formation of *Pp* infection structures rather than stimulating or priming the plant immune system for defence. Therefore, scopoletin may qualify as a natural fungicide to fight SBR.

## Results

### The phenylpropanoid pathway and scopoletin biosynthesis are triggered in the Arabidopsis non‐host during post‐invasion defence of *Pp*

**Figure 1:**
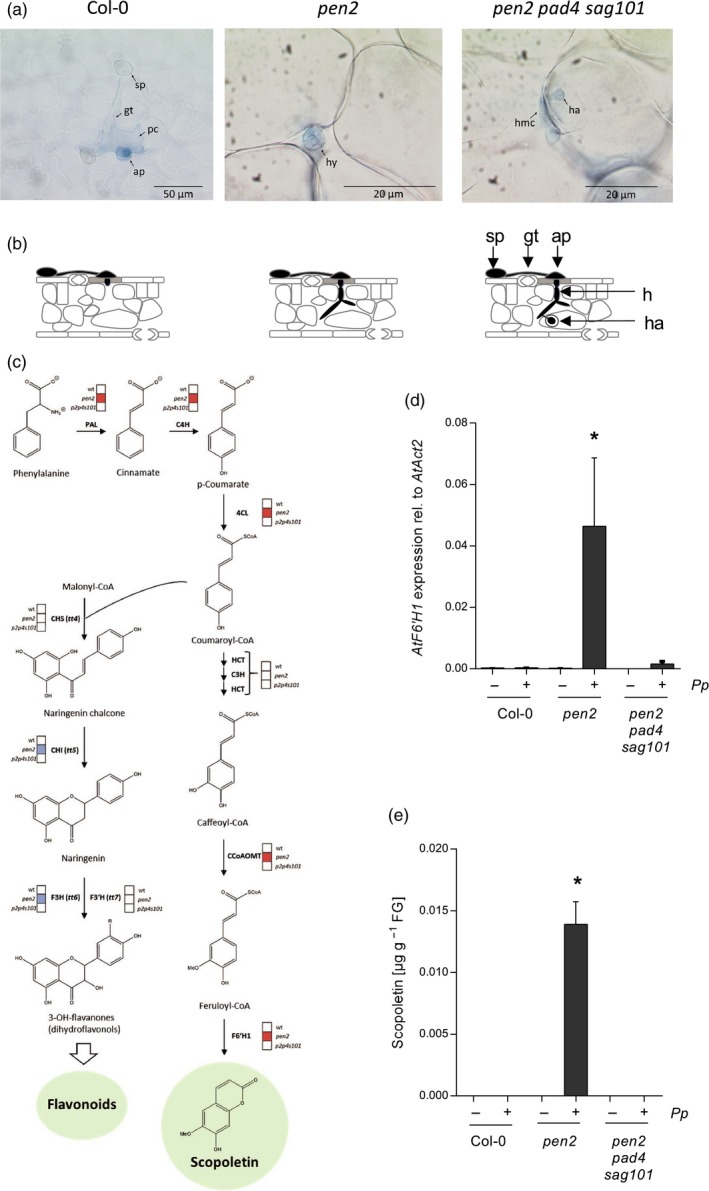
Scopoletin biosynthesis is induced in Arabidopsis during post‐invasion non‐host defence of *Pp*. (a) Micrographs of representative interaction sites of *Pp* with Arabidopsis wild‐type (wt), *pen2*, and *pen2 pad4 sag101* plants at 2 days after inoculation with uredospores. Leaves were subjected to trypan blue staining and inspected by bright‐field microscopy. sp, uredospore; gt, germ tube; ap, appressorium; pc, penetrated cell; hy, intercellular growing hypha; hmc. haustorial mother cell; ha, haustorium. (b) Schematic depiction of representative *Pp* interactions with different Arabidopsis genotypes. Black, fungal structures; grey, dying plant epidermal cell. (c) Microarray analysis revealed significantly (*P* < 0.05) increased (red box) mRNA abundance of PPP genes for scopoletin biosynthesis, yet unaltered (white box) or repressed (blue box) expression of flavonoid biosynthesis genes in the Arabidopsis *pen2* mutant 2 days after inoculation with *Pp* relative to mock‐treated (0.01% (v/v) Tween‐20) controls. These changes in PPP gene activity are absent in the wild‐type or the *pen2 pad4 sag101* triple mutant. Expression values are derived from three independent experiments, each comprising pools of four plants per treatment and genotype. *F6′H1* mRNA (d) and scopoletin accumulation (e) were confirmed by RT‐qPCR and high‐performance liquid chromatography analysis, respectively, in three additional experiments. *F6′H1 t*ranscript abundance was normalized to *ACT2* as the reference gene. In each experiment, RNA and scopoletin were extracted from 5 g of pooled leaves (~12 plants) per genotype and treatment. Shown are the average values and standard deviation of three independent experiments. Asterisks indicate significant differences to the adequate mock control (Holm–Sidak's multiple comparisons test; *P* < 0.01).

To identify metabolites that are specifically associated with post‐invasion resistance of Arabidopsis to *Pp* and which, therefore, may have potential for use in plant protection we inoculated the Arabidopsis wild‐type and the *pen2* and *pen2 pad4 sag101* triple mutant with *Pp*. In the Arabidopsis wild‐type, *Pp* is most frequently arrested in the penetrated epidermal cell (Figure 1a,b). Trypan blue staining indicates that this usually is associated with death of the attacked cell (Figure 1a,b). Fungal invasion of the wild‐type was rarely observed. In contrast, intercellular hyphae (Figure 1a,b) are frequently seen in the mesophyll of the *Pp*‐inoculated *pen2* mutant which lacks preinvasion NHR. Because haustoria only sporadically develop in *pen2* but frequently in *pen2 pad4 sag101* (Figure 1a,b), post‐invasion NHR is intact in the *pen2* but absent from the *pen2 pad4 sag101* mutant (Langenbach *et al*., 2013).

We then assayed the expression of genes in the three genotypes using DNA microarray and MapMan analysis. This approach disclosed that genes in the PPP were preferably expressed in the *Pp*‐inoculated *pen2* mutant with post‐invasion resistance to SBR (Figures 1c and S1 and Tables S1–S3). Activation of those genes was absent, or only low, in the preinvasion‐resistant wild‐type and the *pen2 pad4 sag101* mutant with impaired pre‐ and post‐invasion resistance to SBR (Langenbach *et al*., 2013) (Figures 1c and S1 and Table S1). We hence concluded that the *Pp*‐induced activation of *PPP* genes is specifically triggered during post‐invasion NHR to SBR in Arabidopsis. Notably, preinvasion defence in the wild‐type and post‐invasion defence in *pen2* did not include activation of genes with a role in the biosynthesis of glucosinolates (Figure S1 and Table S2), which are the substrates for PEN2‐mediated defence of Arabidopsis against infectious fungi (Bednarek *et al*., 2009). PPP‐associated genes whose expression was specifically induced in the *Pp*‐inoculated *pen2* mutant were those encoding PHENYLALANINE AMMONIA LYASE (PAL), CINNAMATE‐4‐HYDROXYLASE (C4H), and 4‐COUMARATE:CoA LIGASE (4CL) (Figure 1c and Table S1). These enzymes catalyze the three initial steps of the PPP (Fraser and Chapple, 2011). Of the genes encoding enzymes acting downstream of coumaroyl‐CoA in the PPP, *Pp*‐induced expression was mainly limited to those in the coumarin/lignin/sinapate ester branch. In contrast, the expression of chalcone/flavonoid biosynthesis genes, such as those encoding CHALCONE SYNTHASE (CHS), CHALCONE ISOMERASE (CHI), FLAVANONE 3‐HYDROXYLASE (F3H; TRANSPARENT TESTA 6; TT6) and FLAVONOID 3′‐HYDROXYLASE (F3′H; TRANSPARENT TESTA7; TT7) was slightly or markedly reduced upon inoculation with *Pp* (Figure 1c and Tables S1 and S2). Genes encoding HYDROXYCINNAMOYL‐CoA SHIKIMATE:QUINATE HYDROXYCINNAMOYL TRANSFERASE (HCT) and COUMAROYL SHIKIMATE 3′‐HYDROXYLASE (C3′H; REDUCED FLUORESCENCE8; REF8) were active at medium‐to‐high level in all genotypes, whether inoculated or not (Table S1). Both enzymes are needed for the conversion of coumaroyl‐CoA to caffeoyl‐CoA which is required for the biosynthesis of scopoletin, lignin and sinapate esters (Kai *et al*., 2008; Fraser and Chapple, 2011). The key enzyme of scopoletin biosynthesis, F6′H1, catalyzes the ortho‐hydroxylation of feruloyl‐CoA to 6‐hydroxy‐feruloyl‐CoA which can be spontaneously converted to scopoletin (Kai *et al*., 2008). Among the *PPP* genes whose expression we analyzed, *F6′H1* showed the strongest increase in *Pp*‐induced expression (~15‐fold) (Table S1). Post‐invasion defence‐associated activation of this gene in the *pen2* mutant was confirmed by quantitative reverse transcription PCR analysis (RT‐qPCR) (Figure 1d).

To validate whether the enhanced expression of *F6′H1* during post‐invasion defence of Arabidopsis would correlate with increased scopoletin biosynthesis, we analyzed the accumulation of scopoletin in leaves of the three above‐mentioned Arabidopsis genotypes upon inoculation with *Pp*. Consistent with the abundance of *F6′H1* mRNA, high‐performance liquid chromatography (HPLC) analysis disclosed the accumulation of scopoletin in leaves of the *Pp*‐inoculated *pen2* mutant (Figure 1e). Microscopic analysis was apparently not sensitive enough to reveal scopoletin fluorescence in the proximity of autofluorescent invading hyphae (Figure S2). Scopoletin was absent from the wild‐type and the *pen2 pad4 sag101* mutant no matter whether inoculated with *Pp* or not (Figure 1e). These findings suggest that in *Pp*‐inoculated Arabidopsis, scopoletin is specifically synthesized during post‐invasion NHR.

### Scopoletin is absent from leaves of both SBR‐susceptible and resistant soybean cultivars

**Figure 2:**
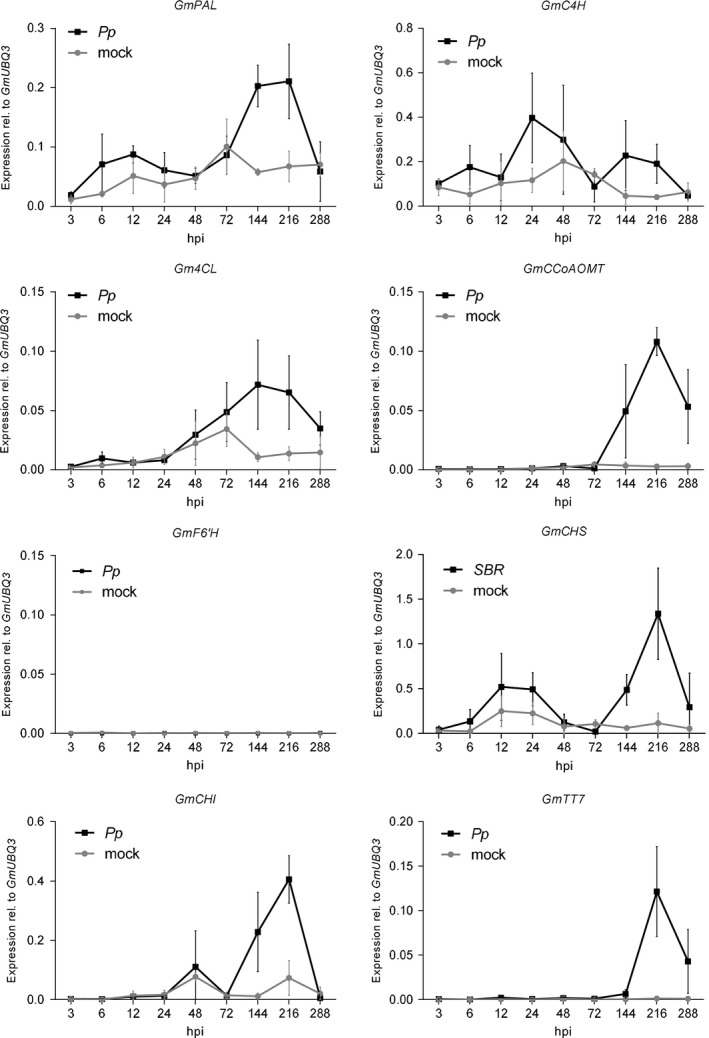
*Pp* infection of soybean correlates with increased expression of genes associated with flavonoid but not scopoletin biosynthesis. Gene expression was determined by RT‐qPCR at the indicated time points after inoculation of the SBR‐susceptible soybean cultivar W82 with *Pp* uredospores suspended in 0.01% (v/v) Tween‐20 () or mock inoculation with 0.01% (v/v) Tween‐20 (). Expression of the indicated genes of interest was normalized to *GmUBQ3* transcript abundance. The average values and standard deviation of three independent experiments are shown. For each treatment and time of sampling, RNA was extracted from two pooled trifoliate leaves of different soybean plants.

To compare the activation pattern of PPP routes in the Arabidopsis non‐host and soybean host we first monitored the expression of genes potentially involved in scopoletin and flavonoid biosynthesis in the SBR‐susceptible soybean cultivar Williams 82 (W82) after inoculation with the highly virulent *Pp* isolate BR05 (Langenbach *et al*., 2016b). Consistent with an earlier report (Schneider *et al*., 2011) we detected biphasic induction of PPP genes in the soybean *PAL*,* C4H* and *CHS* families with an early expression peak at 12–24 h post inoculation (hpi) and a late expression maximum at 144–216 hpi (Figure 2). Activation of most other soybean PPP genes whose expression we analyzed was induced only during the late expression period (Figure 2). *Pp*‐induced activation of *PAL*,* C4H* and *4CL* genes in both Arabidopsis (Figure 1) and soybean (Figure 2) suggests species‐independent activation of PPP genes for enzymes upstream of coumaroyl‐CoA, the major branching point of flavonoid and coumarin biosynthesis in higher plants. However, different from Arabidopsis (Figure 1c), genes belonging to families of the flavonoid biosynthesis branch (*CHS*,* CHI* and *F3′H*) were increasingly expressed in *Pp*‐inoculated soybean whereas scopoletin biosynthesis was not triggered in this plant. Despite *CAFFEOYL‐CoA METHYLTRANSFERASE* (*CCoAOMT*) mRNA accumulating at ~216 h post infection, neither *GmF6′H1* (Glyma.03g096500) nor *GmF6′H2* (Glyma.07g124400) mRNA was detected in mock‐ or *Pp‐*inoculated soybean leaves (Figures 2 and S3a,b). Consistently, scopoletin did not accumulate in soybean leaves at any time after *Pp*‐ or mock inoculation (Figure S3c,d).

To analyze the activation of the PPP and the accumulation of scopoletin in an incompatible *Pp*‐soybean interaction, we inoculated the SBR‐resistant soybean genotype Ji wo dou (PI 594754) with the same *Pp* isolate that was used in the above experiments. In contrast to its compatible interaction with W82, *Pp* isolate BR05 fails to develop uredosori and sporulate on Ji wo dou. It rather induces the formation of reddish brown (RB) lesions (Figure S4a) that are characteristic for an incompatible soybean‐*Pp* interaction (Pham *et al*., 2009; Pandey *et al*., 2011). RT‐qPCR analysis revealed strong activation of *GmPAL* and *GmCHS* early after *Pp* BR05 inoculation in Ji wo dou (Figure S4b). Transcript abundance of both genes increased far stronger 12 and 24 h after inoculation of the resistant cultivar with *Pp* (Figure S4b) than in the compatible interaction of *Pp* with W82 (Figure 2) For example, *GmPAL* was induced 4.8‐fold and 6.6‐fold in the resistant cultivar at 12 and 24 hpi (Figure S4b) while its expression raised only 1.7‐fold relative to the mock control at both time points in the susceptible interaction (Figure 2). A second increase in expression (144–288 hpi) of PPP‐linked genes was not observed in the incompatible interaction (Figure S4b). In contrast to *GmPAL* and *GmCHS*, neither *GmF6′H1* nor *GmF6′H2* mRNA was detected at any assayed time point after inoculation in Ji wo dou (Figure S4b). Because scopoletin was not detectable in Ji wo dou (Figure S4) or in the susceptible accession W82 (Figure S3) scopoletin biosynthesis is neither triggered in resistant nor susceptible cultivars of the soybean host upon inoculation with *Pp*. Our data indicate that *Pp* inoculation activates divergent metabolic routes in its host and non‐host plants that is flavonoid biosynthesis in soybean and coumarin synthesis in Arabidopsis.

### Scopoletin inhibits the development of *Pp* pre‐infection structures

**Figure 3:**
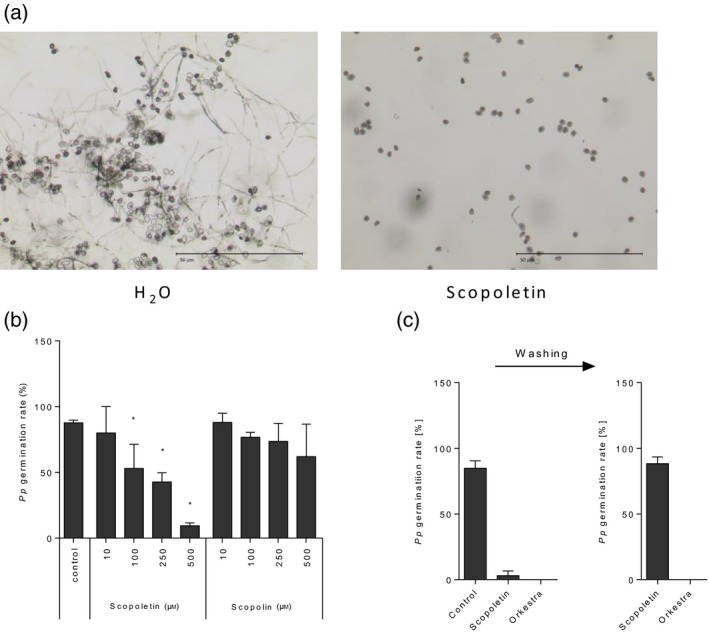
Scopoletin but not scopolin reversibly suppress *Pp* germination *in vitro*. (a) Microscopic images of *Pp* uredospores at 16 h after incubation in the absence or presence of 500 μm scopoletin. Scale bar: 50 μm. (b) Germination rates were determined microscopically 16 h after incubation of *Pp* uredospores on glass slides in the presence of the indicated concentrations of scopoletin, scopolin, or water (control). Average values and standard deviation are derived from data of three independent experiments. Asterisks indicate significant differences to the control (Holm–Sidak's multiple comparisons test; *P* < 0.05). (c) Germination of *Pp* uredospores is completely reconstituted upon washing of scopoletin but not Orkestra‐treated spore suspensions. *Pp* uredospore germination was evaluated 16 h after incubation in 500 μm scopoletin, a 1:2000 dilution of Orkestra (left panel) or water (control). After washing, spores were incubated for another 4 h in water and germination frequencies assayed again (right panel). Spore suspensions were always supplemented with 0.01% (v/v) Tween‐20. the average values and standard deviation from three independent experiments are shown. At least 100 spores were counted per condition in a single experiment.

**Figure 4:**
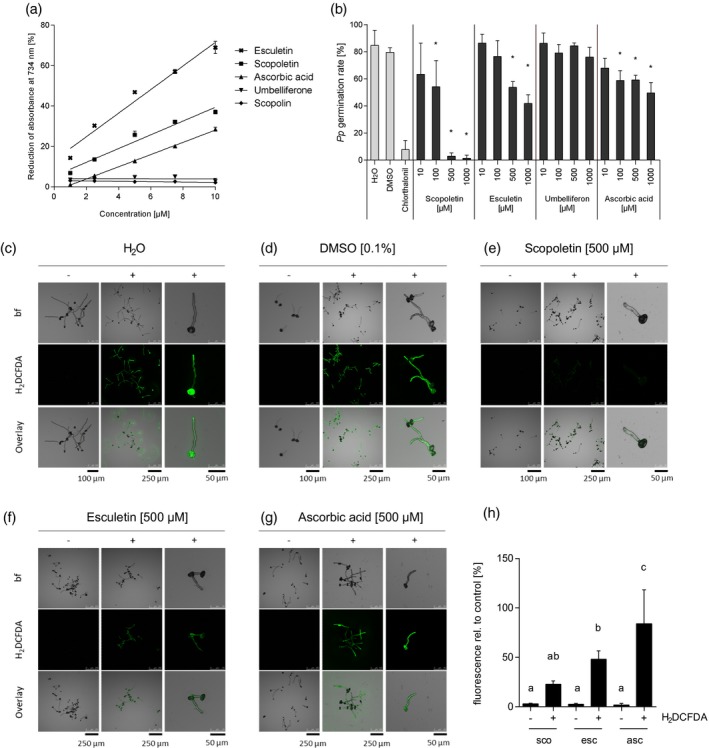
Suppression of *Pp* germination correlates with reduced fungal ROS accumulation in pre‐infection structures. (a) Esculetin (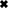) and scopoletin (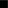) but not scopolin (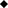) or umbelliferone (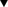) have ROS‐scavenging activity. Antioxidant capacity of the coumarins was determined in an ABTS assay and compared with ascorbic acid (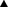) as an antioxidant reference. Reduction of ABTS∙+ radicals by antioxidants correlates with a reduction of absorbance at 734 nm. Shown are mean values and SD from three independent experiments. (b) Non‐antioxidant umbelliferone is incapable of inhibiting *Pp* germination whereas ROS scavengers esculetin and ascorbic acid inhibit *Pp* development. However, they are far less fungistatic than scopoletin. Germination rate of at least 100 *Pp* uredospores was determined microscopically 16 h after incubation on polyethylene foil in the presence of different coumarins or ascorbic acid at different concentrations. Water was used as a negative control for ascorbic acid treatment and 0.1% DMSO served as negative control for treatments with scopoletin, esculetin and umbelliferone, all coumarin samples containing 0.1% DMSO. The contact fungicide chlorothalonil (15 μg ml^−1^ in 0.01% DMSO) was included as a known inhibitor of *Pp* germination. Average values and SD from three independent experiments are shown. Asterisks indicate significant differences to the appropriate negative control (Holm–Sidak's multiple comparisons test; *P* < 0.05). Histochemical H_2_DCFDA staining discloses ROS production in germinated *Pp* uredospores and germ tubes and intracellular ROS‐scavenging capacity of different coumarins and ascorbic acid. Spores were incubated in water for 3 h to allow for germination and then supplemented with water (c), 0.1% DMSO (d) or 500 μm of each, scopoletin (e), esculetin (f) or ascorbic acid (g). After 1 h, H_2_DCFDA was added to a final concentration of 2.5 μg ml^−1^ and incubated for 30 min. ROS accumulation was analyzed by monitoring the fluorescence intensity upon H_2_DCFDA staining in a confocal laser scanning microscope (488 nm excitation and 500–550 nm emission). (h) For semi‐quantitative determination of fungal ROS accumulation maximum H_2_DCFDA fluorescence was determined in >100 *Pp* germ tubes in at least three independent experiments. Shown is the average maximum H_2_DCFDA fluorescence in treated germ tubes relative to the respective control. Groups a, b and c are significantly different from each other in one‐way analysis of variance (anova) (Holm–Sidak method, *P* < 0.05).

To evaluate whether scopoletin influences *Pp* development we next investigated its antifungal activity *in vitro*. As shown in Figure 3(a,b), scopoletin inhibited the germination of *Pp* spores in a concentration‐dependent manner (ED_50_ ~190 μm). At a concentration of 100 μm, scopoletin significantly reduced spore germination while at 500 μm it almost completely abolished fungal development (Figures 3b,c and 4b). In contrast to scopoletin, its glycosylated form scopolin did not significantly affect the germination of *Pp* uredospores at any concentration tested (Figure 3b).

Next, we assayed whether the inhibition of spore germination by scopoletin would be reversible or not. To do so, we first compared *Pp* uredospore germination in the absence or presence of scopoletin or the commercial fungicide Orkestra (a mixture of the fungicides pyraclostrobin and fluxapyroxad). The latter is used to control SBR in the field (Juliatti *et al*., 2017) and served as a positive control in this assay. We then thoroughly washed the spores that were treated with scopoletin or Orkestra and scored the recovery of fungal germination after incubation for another 4 h in water. As shown in Figure 3(c), the germination of *Pp* spores was inhibited by ~96 and 100% in the presence of scopoletin or Orkestra. Thorough washing of the scopoletin‐treated uredospores restored spore germination, which remained suppressed upon washing the Orkestra‐treated spores (Figure 3c). Therefore, in contrast with Orkestra, scopoletin seems to exert fungistatic rather than fungicidal activity against *Pp*.

### Suppression of *Pp* spore germination by scopoletin is associated with reduced accumulation of reactive oxygen species in *Pp* pre‐infection structures

Development and virulence of plant pathogenic fungi often require endogenous ROS accumulation (Egan *et al*., 2007). Because scopoletin is an antioxidant, we reasoned that the coumarin might scavenge ROS, thereby inhibiting fungal growth and development. Therefore, we investigated whether ROS would accumulate in germinating spores and germ tubes of *Pp* using histochemical ROS staining with 2′,7′‐dichlorodihydrofluorescein diacetate (H_2_DCFDA). Indeed, ROS were present in germinating spores and fungal germ tubes with increasing fluorescence towards the distal tip of the growing germ tube (Figure S5a). When *Pp* pre‐penetration structures were subjected to Coomassie Brilliant Blue staining, we found that the polar increase in fluorescence coincided with the density of the cytoplasm. The latter migrates along with growing germ tubes and has a higher density at the germ tube tips (Figure S5b). To address whether ROS also accumulate in *Pp* infection structures *in planta* we subjected *Pp*‐inoculated leaves of the various Arabidopsis genotypes to staining with 3,3′‐diaminobenzidine (DAB), which polymerizes directly upon contact with H_2_O_2_ (Thordal‐Christensen *et al*., 1997). Figure S6 shows that, in addition to penetrated plant cells (Figure S6a) (Loehrer *et al*., 2008), invasive hyphae in the mesophyll of *pen2* (Figure S6b) and fungal haustoria in *pen2 pad4 sag101* (Figure S6c) were stained by DAB. Because *Pp* infection structures were also stained in the *rbohD* mutant which lacks a functional NADPH oxidase and does not accumulate ROS upon *Pp* penetration (Figure S6d,e), DAB staining of fungal hyphae is likely due to fungal, but not plant‐derived ROS. Because ROS were detected in *Pp* at all developmental stages analyzed (Figures S5 and S6) we hypothesize that ROS accumulation might be important to proper *Pp* development and that the antioxidant activity of scopoletin may cause the observed germination arrest. Therefore, we next compared the antioxidant capacity of scopoletin, scopolin, the structurally related coumarins umbelliferone (7‐hydroxycoumarin) and esculetin (6,7‐dihydroxycoumarin), as well as the known ROS scavenger ascorbic acid with regard to their efficacy to inhibit *Pp* spore germination. Different from scopoletin, neither scopolin nor umbelliferone displayed antioxidant activity in an 2,2′‐azino‐bis (3‐ethylbenzothiazoline‐6‐sulphonic acid) (ABTS)‐based assay (Figure 4a). They also did not interfere with fungal differentiation at any concentration tested (Figures 3b and 4b). Esculetin exerted higher antioxidant activity than scopoletin (Figure 4a) but was less effective at inhibiting *Pp* spore germination (Figure 4b). *In vitro* development of pre‐infection structures was inhibited by only ~35% when spores were incubated in the presence of 500 μm esculetin and inhibition only slightly increased at 1 mm esculetin. In contrast, fungal germination was almost completely suppressed when spore suspensions were supplemented with 500 μm scopoletin. Similarly, the known oxygen radical scavenger ascorbic acid exerted only slightly lower antioxidant activity than scopoletin (Figure 4a), but was far (>10‐fold) less active than scopoletin at antagonizing *Pp* development at concentrations of 500 μm and more (Figure 4b). This result initially suggested another mode of action of scopoletin in *Pp* inhibition, alternative or in addition to its ROS‐scavenging activity. However, in close correlation to its ability of inhibiting *Pp* germination, scopoletin suppressed intracellular ROS production in *Pp* pre‐infection structures to a much higher extent than ascorbic acid or esculetin (Figure 4c–h). When compared with the controls, H_2_DCFDA staining decreased by ~80% in the presence of scopoletin whereas fluorescence was reduced by only ~15 and ~50% upon treatment with ascorbic acid or esculetin (Figure 4h). The data therefore demonstrate a close correlation between the ability of a tested compound to inhibit intracellular ROS accumulation during *Pp* germination and its antifungal activity.

### Scopoletin provides protection by inhibiting *Pp* development rather than activating plant defence

**Figure 5:**
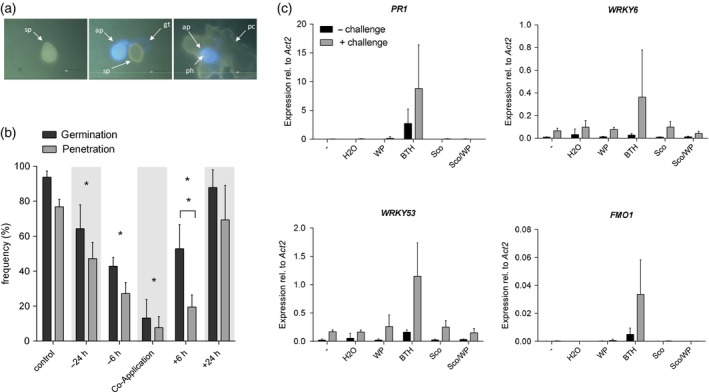
Scopoletin suppresses *Pp* infection structure formation on the Arabidopsis leaf surface but does not activate or prime the expression of defence‐related genes. (a) Representative images of *Pp* interaction sites with Arabidopsis in destained leaves 2 days after inoculation showing an ungerminated uredospore (sp) (left), a germinated spore with germ tube (gt), an appressorium (ap) that has not penetrated (center), and a penetration event with fungal spore, appressorium, penetration hyphae (ph) and autofluorescent epidermal plant cell (collapsed cell, cc) (right). Autofluorescent fungal structures and penetrated plant cells were visualized by epifluorescence microscopy. (b) *Pp* germination and penetration frequencies on Arabidopsis leaves treated with 500 μm scopoletin either before (−24 h, −6 h), or after (+6 h, +24 h) inoculation or upon co‐application of uredospores with scopoletin or water (control). Leaves were collected 2 dpi, destained on chloral hydrate‐soaked paper towels and analyzed by fluorescence microscopy. For each treatment >100 interaction sites on two or more leaves of two plants were evaluated and assigned to each of the above described interaction categories. Asterisks indicate significant differences in germination and penetration rates compared to the control (Holm–Sidak's multiple comparisons test; *P* < 0.05). Asterisks above a bracket indicate significant differences in the germination to penetration ratio after a certain treatment (Student's *t*‐test; *P* < 0.05). Shown are the average values and SD of three independent experiments. (c) Arabidopsis plants were pretreated with water, wettable powder (WP), 100 μm of the salicylic acid mimic benzo(1,2,3)thiadiazole‐7‐carbothioic acid S‐methyl ester (BTH) in WP, 500 μm scopoletin (Sco), 500 μm scopoletin in wettable powder (Sco/WP) or left untreated (−). Three days after pretreatment, two leaves of half of the plants were challenged by water infiltration. 3 h after challenge, the challenged leaves and two leaves of unchallenged plants were harvested and analyzed for the expression levels of the defence marker genes *PR1*,* FMO1*,* WRKY6* and *WRKY53* by RT‐qPCR. Expression of these genes was normalized to *ACTIN2* as the reference gene. In each experiment, leaves derived from three different plants per treatment were analyzed. Shown are the average values and SD from two independent experiments.

To investigate whether exogenously applied scopoletin has plant‐protecting activity we first took advantage of the interaction of *Pp* with Arabidopsis. Using Arabidopsis facilitated microscopic analysis because autofluorescent fungal infection structures and penetrated epidermal plant cells (Figure 5a) can be monitored more easily and accurately in Arabidopsis than in soybean. No histochemical staining is needed which may wash‐off ungerminated spores from the plant and therefore prohibit precise microscopic assessment of the *Pp*−plant interaction. Consistent with its fungistatic activity *in vitro* (Figures 3 and 4b), scopoletin upon spray application reduced *Pp* spore germination on the Arabidopsis leaf surface and the subsequent penetration of epidermal cells (Figure 5b). Co‐application of scopoletin and fungal spores was most effective causing a ~10‐fold reduction of fungal spore germination and plant penetration. The protective effect of scopoletin decreased with increasing time between scopoletin treatment and inoculation (Figure 5b). No protection from *Pp* infection was observed when scopoletin was applied 24 hpi, when *Pp* has entered the plant (Loehrer *et al*., 2008). In contrast, scopoletin inhibited both fungal germination and plant penetration at 6 hpi. However, different from all other treatments in this series, plant penetration was significantly more affected than germination when scopoletin was applied at 6 hpi. Compared with mock‐treated controls, spore germination was inhibited by 35% whereas penetration success was reduced by almost 60% (Figure 5b). This indicates that scopoletin not only impairs *Pp* uredospore germination but also the subsequent development of *Pp* structures, which are required for plant penetration.

Next, we tested whether the scopoletin‐mediated protection of Arabidopsis from *Pp* was exclusively caused by its fungistatic activity or whether scopoletin would also trigger endogenous plant defence, either directly or by priming the innate immune response (Conrath *et al*., 2015). We did this by analyzing the expression of known marker genes for pathogen defence in Arabidopsis (Jaskiewicz *et al*., 2011; Conrath *et al*., 2015). In contrast with treatment with a salicylic acid‐mimicking benzothiadiazole, neither direct or primed activation of Arabidopsis defence genes *PATHOGENESIS‐RELATED1* (*PR‐1*), *FLAVIN CONTAINING MONOOXYGENASE1* (*FMO1)*,* WRKY6* or *WRKY53* was observed upon treatment with scopoletin even when applied with a wettable powder carrier (WP) (Figure 5c). Expression of defence marker genes in scopoletin‐pretreated and then either unchallenged or challenged leaves was similar to plants that have not been pretreated or pretreated with water or WP only. These findings suggest that scopoletin provides plant protection by directly inhibiting *Pp* development rather than stimulating the plant immune system.

### Spray application of scopoletin protects soybean from SBR

**Figure 6:**
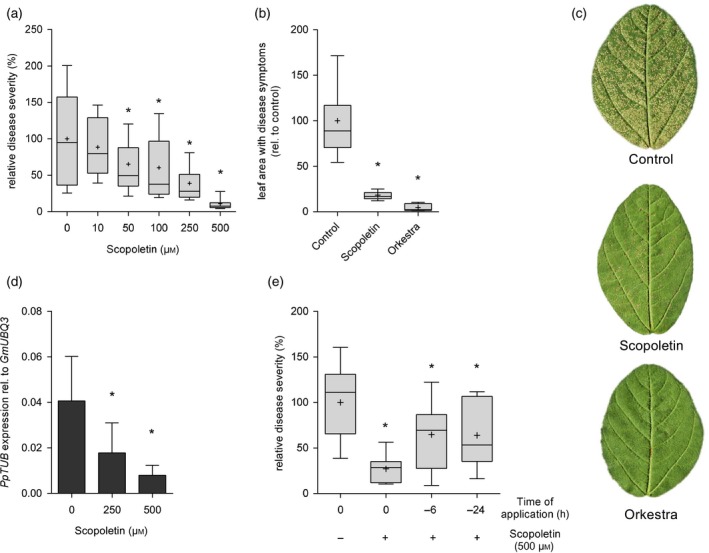
Foliar application of scopoletin protects soybean from SBR. (a) *Pp* uredospore suspensions were supplemented with the indicated concentrations of scopoletin and sprayed on soybean W82 trifoliate leaves. In each experiment, SBR severity was scored by quantifying the relative leaf area with rust symptoms on the first trifoliate leaf of 6–8 different plants at 10 dpi. Shown is the percentage of leaf area with SBR symptoms relative to the control (0 μm scopoletin) from three independent experiments. Data are presented in boxplots with boxes spanning the interquartile range (IQR) and whiskers extending to 1.5*IQR. Average values are represented by a (+) symbol. Asterisks indicate significant differences to controls (Holm–Sidak's multiple comparisons test; *P* < 0.05). (b) Scopoletin is almost as effective as Orkestra in providing protection from SBR when co‐applied with *Pp* uredospores. *Pp* spore suspensions were supplemented without (0 μm, control) or with 500 μm scopoletin or Orkestra (1:2000 dilution) and sprayed on leaves of soybean cultivar W82. Disease severity was scored at 10 dpi by quantifying the relative leaf area with rust symptoms. Shown is the per cent leaf area with SBR symptoms relative to the control (0 μm scopoletin) of three independent experiments. Data are presented in boxplots with boxes spanning the IQR and whiskers extending to 1.5*IQR. Average values are represented by a (+) symbol. Asterisks indicate significant differences from controls (Dunnett's multiple comparisons test; *P* < 0.05). (c) Representative images of leaflets from W82 trifolia which were treated as described in (b). (d) *Pp* mRNA abundance is reduced in leaves treated with scopoletin. W82 plants were sprayed with *Pp* spore suspensions containing 0 μm (control), 250 μm, or 500 μm scopoletin and analyzed at 10 dpi by RT‐qPCR to determine mRNA abundance of *Pp* α*‐TUBULIN* (*PpTUB*) relative to soybean *UBIQUITIN3* (*GmUBQ3*). Experiments were done in triplicate. Significant differences (*P* < 0.05) in Dunnett's multiple comparisons test are marked by asterisks. (e) Pre‐application of scopoletin significantly reduces SBR disease symptoms. Here, 500 μm scopoletin were either sprayed on the leaf surface of W82 soybean 6 h or 24 h prior to inoculation with *Pp* uredospores or co‐applied with spores (0 h; +). As a control, plants were inoculated with a *Pp* spore suspension lacking scopoletin (0 h; −). Disease severity was scored as described above. Average values of three independent experiments are shown. Asterisks indicate significant differences to control (Holm–Sidak's multiple comparisons test; *P* < 0.05).

To assess the potential of scopoletin for use as a natural plant protectant we investigated its capacity to protect soybean from SBR disease. Consistent with its ability to inhibit the formation of *Pp* pre‐infection structures *in vitro* and *in vivo*, scopoletin reduced the susceptibility of soybean to SBR in a concentration‐dependent manner (ED_50_ ~160 μm) (Figure 6a). Co‐application of uredospores and scopoletin at 50 μm was sufficient to significantly reduce SBR symptoms and decrease the diseased leaf area by 35% while at a concentration of 500 μm, scopoletin reduced disease symptoms by ~85% (Figure 6a–c). SBR symptom reduction by ~95% was achieved when spores suspensions were supplemented with Orkestra (Figure 6b,c). Quantification of mRNA of the constitutively expressed *Pp* α‐tubulin gene in inoculated soybean leaves confirmed the reduction in fungal colonization of the host (Figure 6d). Scopoletin, therefore, most likely provides soybean protection from SBR by preventing fungal invasion. Consistent with the findings obtained when analyzing the Arabidopsis−*Pp* interaction, soybean protection was most effective when scopoletin was co‐applied with fungal spores. However, even if scopoletin was applied 6 or 24 h before inoculation in the absence of any adjuvant, the coumarin significantly protected the soybean host from SBR (Figure 6e). These data demonstrate a high potential of scopoletin for use as a fungistatic natural compound for controlling SBR disease.

## Discussion

The vast genetic flexibility of *Pp*, its quick evolution of fungicide insensitivity, its capacity to rapidly overcome gene‐for‐gene resistance in plants and its ability to spread rapidly over long distances (Goellner *et al*., 2010; Twizeyimana *et al*., 2011; Yamaoka, 2014; Simões *et al*., 2018) require a multifaceted SBR management strategy that relies on different modes of action. Due to the limited amount of synthetic fungicide classes in use and only few potential, but not yet applied natural inducers of SBR resistance, we searched for plant metabolites in *Pp*‐resistant Arabidopsis plants that may help fighting SBR. Genome‐wide transcriptome analysis in the Arabidopsis non‐host disclosed activation of scopoletin biosynthesis and repression of flavonoid production during post‐invasion NHR to SBR (Figure 1). In soybean, the flavonoid pathway was activated while scopoletin accumulation was not detected (Figures 2, S3 and S4). The two plant species seem to have evolved different pathways that produce divergent chemicals for their defence. In support of this the phytoalexin camalexin and glucosinolates are major defence‐related compounds in Arabidopsis but absent from soybean and other leguminous plants (Halkier and Gershenzon, 2006; Glawischnig, 2007; Lee *et al*., 2017). Likewise, although widely distributed in the plant kingdom (Gnonlonfin *et al*., 2012) we detected scopoletin in *Pp*‐inoculated Arabidopsis leaves of the *pen2* mutant but neither in uninfected nor infected leaves of the soybean host (Figures 1, S3 and S4). Because of the apparent induction of PPP‐associated genes but inactivity of the two soybean *F6′H1* orthologs in a resistant and a susceptible host cultivar (Figures 2, S3 and S4) and the strong activation of PPP genes including *F6′H1* in the non‐host (Figure 1 and Table S1) we conclude that scopoletin accumulation is tightly associated with *F6′H1* gene expression. The differential response of host and non‐host is unlikely to be caused by lacking recognition of the fungal invader in the host, as no scopoletin was detected even in the incompatible interaction between *Pp* and the resistant cultivar Ji wo dou (Figure S4). The soybean host and Arabidopsis non‐host apparently utilize different chemical repertoires for their defence. Indeed, accumulation of coumarins is thought to be restricted to the roots of soybean (Romani *et al*., 2003). This is most likely different in *Medicago truncatula*, which is considered a non‐host for *Pp*. In contrast with soybean, transcript of an *F6′H1/F6′H2* ortholog (probe set Mtr.38126.1.S1) accumulates in leaves of *M. truncatula* at 12 h after inoculation with non‐adapted *Pp* (Ishiga *et al*., 2015). This indicates that scopoletin may also contribute to SBR defence in another non‐host species that is a closer relative to soybean than Arabidopsis.

However, there has been no indication for scopoletin or other coumarins contributing to the defence of soybean against *Pp*. We, therefore, assume that *Pp* may not have evolved mechanisms for detoxifying coumarins and that scopoletin may be ideal for controlling SBR disease. Similar to its inhibitory activity against other fungi and oomycetes such as *Helminthosporium carbonum*,* Cercospora nicotianae*,* Phytophthora parasitica* var. *nicotianae*,* Puccinia helianthi*,* Sclerotinia sclerotiorum*,* Fusarium oxysporum*, and *Verticillium dahliae* (Tal and Robeson, 1986a; Goy *et al*., 1993; Prats *et al*., 2002, 2006; Gómez‐Vásquez *et al*., 2004; Stringlis *et al*., 2018) scopoletin effectively suppressed the germination of *Pp* uredospores but also reduced fungal penetration when applied after germination (Figures 3, 4b and 5b). This indicates that *Pp* cannot detoxify scopoletin at least at this developmental stage. In contrast with *Pp*, some other fungi have evolved mechanisms for degrading scopoletin (El Oirdi *et al*., 2010; Tal and Robeson, 1986b). Interestingly, the ability to detoxify scopoletin is thought to correlate with the adaptation of a given fungus to a scopoletin‐producing plant (Tal and Robeson, 1986b). For example the sunflower pathogen *Alternaria helianthi* rapidly degrades scopoletin, whereas detoxification of this compound by the non‐adapted fungus *H. carbonum* is much less efficient (Tal and Robeson, 1986b). Our results suggest that this is also true for *Pp*. Due to the absence of the coumarin from its main host soybean no selection pressure may have been imposed on *Pp* to evolve one or more scopoletin‐detoxifying mechanisms.

Several lines of evidence point to intra‐ and extracellular ROS as being required for proper development and virulence of fungi including some plant pathogens (Hansberg *et al*., 1993; Malagnac *et al*., 2004; Egan *et al*., 2007; Heller and Tudzynski, 2011). Our data suggest that germination of *Pp* uredospores and *in planta* differentiation of *Pp* infection structures also involve the intracellular accumulation of ROS by the fungus (Figures 4, S5 and S6). Consistent with a hypothesized requirement of ROS for proper *Pp* development the antioxidant scopoletin but none of the non‐antioxidant coumarins tested (scopolin, umbelliferone) significantly affected *Pp* spore germination (Figures 3b and 4a,b). However, in contrast with the inhibitory effect of antioxidant ascorbic acid on *Magnaporthe grisea* spore germination and appressorium formation (Egan *et al*., 2007), neither the antioxidant ascorbic acid nor the ROS‐scavenging coumarin esculetin inhibited the germination of *Pp* spores as effective as scopoletin (Figure 4a,b). Nevertheless, we detected a close correlation of the capacity of a given compound to suppress intracellular ROS accumulation in *Pp* and its ability to interfere with the germination of *Pp* spores (Figure 4b–h). In fact, like many other organisms, fungi utilize ROS as second messengers to transduce extracellular signals to the nucleus and local ROS bursts are known to contribute to differentiation processes, fungal growth, and virulence (Heller and Tudzynski, 2011). It has been proposed that ROS levels in fungal structures remain low during normal growth whereas differentiation is induced upon local and transient increases in cellular ROS levels (Hansberg and Aguirre, 1990). Differences in the efficacy of suppressing *Pp* spore germination of tested antioxidants may simply be due to their differing membrane passage. Correlations between membrane passage of ROS scavengers and their biological activity are known for other fungi. For example only water‐soluble, low‐molecular‐weight antioxidants that easily enter a cell suppressed *Neurospora crassa* differentiation (Hansberg *et al*., 1993). Compared with esculetin, a better membrane passage of the membrane‐permeable and less hydrophilic coumarin scopoletin (Galkin *et al*., 2009; Sarpietro *et al*., 2011) may compensate for its lower antioxidant activity and lead to increased ROS scavenging in fungal pre‐infection structures and higher efficacy in suppressing *Pp* germination. However, we cannot exclude that the scopoletin‐associated reduction in intracellular ROS levels is not causative for the arrest of spore germination, but simply a side effect resulting from the uptake of the antioxidant coumarin by fungal spores and germ tubes. Scopoletin may also act via alternative or additional pathways. However, because spore germination was efficiently inhibited by scopoletin in the dark, the mode of action is most probably independent of excited‐state proton‐transfer reactions, this has been proposed for hydroxycoumarins when counteracting fungal infection (Simkovitch and Huppert, 2015). Scopoletin also neither directly influences the pH value nor buffers alkalinization by *Pp* uredospores (Figure S7), which is known to contribute to fungal virulence (reviewed by Vylkova, 2017). Yet, similar to the known effects of scopoletin and some other coumarins on eukaryotic cells, scopoletin might affect fungal cell wall structure, or interfere with fungal sterol biosynthesis or activation of cellular signalling cascades (Pan *et al*., 2009; Guerra *et al*., 2015; Ayine‐Tora *et al*., 2016), thereby inhibiting fungal germination. Independent of the mechanism by which scopoletin suppresses *Pp* spore germination, our results show that the plant‐protective effect of scopletin is exclusively based on its fungistatic activity. It does not involve direct or primed activation of plant defence (Figure 5c). This separates scopoletin from other known plant protectants such as silicon, phosphite, and the isothiocyanate sulforaphane. The latter two compounds suppressed *Pp* development directly, but also induced some defence genes while they primed others for enhanced activation (Gill *et al*., 2018; Schillheim *et al*., 2018). Silicon likely provides SBR resistance by stimulating the PPP or by establishing a penetration barrier in the plant cuticle (Ma and Yamaji, 2006; da Cruz *et al*., 2013) without directly interfering with fungal development. Interestingly, a directly suppressing effect against *Pp* such as the one observed here for scopoletin, has been reported for other NHR‐associated phytoalexins such as medicarpin and ononin (Ishiga *et al*., 2015).

When co‐applied with *Pp* spores to soybean leaves, scopoletin at 500 μm protected soybean from SBR nearly as effectively as Orkestra (Figure 6). The coumarin therefore seems to have good application potential for fighting *Pp* even though the efficacy of SBR control was lower when scopoletin was applied prior to inoculation (Figure 6e). Upon co‐application uredospores are suspended in a solution of scopoletin. Therefore, spores are entirely surrounded by the coumarin which results in efficient inhibition of germination. In contrast, pre‐application of an aqueous scopoletin solution to soybean leaves resulted in droplet formation and incomplete coverage of the hydrophobic and trichome‐rich soybean leaf surface. This may have caused unequal coverage of the leaf surface and may therefore have attenuated the protective effect of scopoletin. Addition of an appropriate formulation may significantly improve leaf coverage and disease control efficacy of scopoletin. For example, more than 50% improved control of bean rust was achieved by supplementing the contact fungicide maneb with commercial adjuvants (Gent *et al*., 2003). Despite being more photostable than related coumarins such as esculetin (Smith *et al*., 2010) foliar application of scopoletin may result in light‐induced degradation of the compound. However, sunflowers secrete a blend of coumarins, which include scopoletin, and deposit them on the surface of their leaves to avoid rust disease (Prats *et al*., 2002, 2007). In practice, the possible photodegradation of scopoletin may be avoided by encapsulation in biodegradable nanocontainers (e.g. cyclodextrins), as achieved for other PPP metabolites (Kfoury *et al*., 2016). In addition, employing surfactants and spreaders would certainly help reducing surface tension, increase leaf coverage, and improve the efficacy of scopoletin as a plant protectant. In summary, scopoletin represents a promising natural fungicide that may be utilized to complement current SBR management strategies. Besides its plant‐protecting capacity, scopoletin is also known for its diverse human health‐promoting effects and its abundance in frequently consumed vegetables such as sweet potato or cassava (Gnonlonfin *et al*., 2012). Scopoletin also is present on the leaf surface of widely grown crops such as sunflower (Prats *et al*., 2002, 2007), indicating a rather low risk for causing unintended negative sanitary or ecological side effects upon its utilization in agriculture.

Besides its potential use for spray application, it will be interesting to analyze whether scopoletin‐accumulating, transgenic soybean lines have reduced susceptibility to SBR. It is tempting to speculate, that *in planta*‐accumulating scopoletin may scavenge ROS in fungal infection structures in the mesophyll (Figure S6) and, thereby, interfere with hyphal growth or, for example, differentiation of fungal haustoria that are required for establishing a biotrophic interaction with the host. It is currently unknown whether *in planta*‐accumulating scopoletin would affect fungal differentiation. However, our data confirm a protective role of scopoletin when deposited on the leaf surface (Figure 6). Therefore, an engineering approach for the targeted secretion of scopoletin to the phylloplane appears to be the most promising strategy. In support of this, Dobritzsch *et al*. (2016) disclosed that engineered export of *in planta* synthesized hydroxycinnamic acid amides to the leaf surface correlates with decreased germination of *P. infestans* spores and reduced growth of the oomycete in transgenic potato lines. We are currently generating transgenic soybean lines expressing combinations of *AtF6′H1*, transcriptional activators of *PPP* genes, and scopoletin exporters under control of tissue‐specific promoters. This should enable us to evaluate the feasibility of the genetic‐engineering approach for providing enhanced SBR resistance.

## Experimental procedures

### Plant and fungal material

Arabidopsis wild‐type plants (Col‐0), and the *pen2*, the *pen2 pad4 sag101* and the *rbohD* mutant were grown as described (Langenbach *et al*., 2013). Soybean plants [(*Glycine max* cultivar Williams 82 (PI 518671) and Ji wo dou (PI 594754)] were grown in a chamber in long day at 16 h photoperiod and 8 h dark at 24°C and 20°C, respectively. *Pp* isolate (BR05) was maintained on susceptible soybean plants cultivar Abelina. Three‐week‐old soybean plants were spray‐inoculated using an atomizing sprayer (Carl‐Roth, https://www.carlroth.com) with a 1 mg ml^−1^ *Pp* spore suspension supplemented with 0.01% (v/v) Tween‐20. After incubation at high humidity for 16 h and in the dark, plants were kept as described above. Uredospores were collected from infected soybean leaves starting at ~14 dpi.

### Plant inoculation

For inoculation of Arabidopsis and soybean, *Pp* uredospores were harvested from severely infected soybean plants and suspended in water/0.01% (v/v) Tween‐20 at a density of 1 mg ml^−1^ (soybean) or 1.5 mg ml^−1^ (Arabidopsis). Plants were inoculated by spraying spore suspensions using an atomizing sprayer, incubated for 16 h at high humidity and then transferred to normal growth conditions until analysis.

### DNA microarray analysis

Two days after treatment with 0.01% (v/v) Tween‐20 (mock) or upon inoculation with a *Pp* spore suspension in 0.01% (v/v) Tween‐20, leaves were harvested from Arabidopsis wild‐type, *pen2*, and the *pen2 pad4 sag101* mutant and used for RNA extraction or subjected to trypan blue staining. As verified by microscopic analysis of trypan blue‐stained leaves (Langenbach *et al*., 2013), the fungus at this time had already penetrated the epidermis and/or established hyphae and/or haustoria in the mesophyll in the different Arabidopsis accessions. This indicates normal colonization of the plant by the fungus. Per genotype and treatment, four severely infected leaves were pooled prior to RNA extraction. Total RNA was extracted as described (Chomczynski and Sacchi, 1987) until chloroform extraction. Following centrifugation, the aqueous phase was not precipitated with isopropanol but rather transferred to RNeasy Mini Spin Columns (Qiagen, https://www.qiagen.com). RNA was washed and eluted as described by the manufacturer. Only RNA with RNA integrity number values >8 were used for subsequent Affymetrix ATH1‐gene chip analysis at the IZKF Münster, Germany. Raw data from three independent experiments were processed using ROBINA (Lohse *et al*., 2012) for identifying differentially expressed genes among mock‐treated and inoculated genotypes. Data were normalized using the robust multi‐array average method (Irizarry *et al*., 2003). Multiple testing for each comparison was done separately and *P*‐values corrected by the Benjamini−Hochberg (BH) procedure. The *P*‐value cutoff for differentially expressed genes was set to 0.05. MAPMAN was utilized to visualize the activation or repression of genes in different metabolic pathways (Thimm *et al*., 2004).

### RT‐qPCR analysis

To determine the abundance of transcripts of genes of interest (GOI), RNA from Arabidopsis was extracted as described (Chomczynski and Sacchi, 1987). RNA from soybean leaves was extracted by mixing about 100 mg frozen, thoroughly ground leaves with 555 μl lysis buffer (2% (w/v) ultrapure sodium dodecyl sulphate (SDS), 68 mm tri‐sodium citrate, 132 mm citric acid, 10 mm EDTA, pH ~3.5). For protein precipitation, 185 μl of protein precipitation buffer (4 m NaCl, 17 mm tri‐sodium citrate, 33 mm citric acid, pH ~3.5) was added and samples incubated for 5 min on ice. After centrifugation for 10 min at room temperature (RT), the supernatant was transferred in a fresh test tube and RNA precipitated by adding 550 μl of 2‐propanol and incubation for 15 min at RT. Samples were centrifuged for 5 min at RT and pellets washed with 500 μl ethanol before being dried on ice in a fume hood. RNA was dissolved in 30 μl RNase‐free water. RNA was transcribed to cDNA using 9‐mer random oligonucleotides and RevertAid reverse transcriptase (ThermoFisher Scientific, https://www.thermofisher.com) as described by the manufacturer. Transcript abundance was quantified in a BIO‐RAD CFX384 REAL‐TIME System with iTaq^™^ Universal SYBR^®^ Green Supermix (BIO‐RAD, http://www.bio-rad.com/) as described by the manufacturer. RT‐qPCR was performed as described (van de Mortel *et al*.,^2007^; Langenbach *et al*., 2013). Primers were designed according to standard criteria (Udvardi *et al*., 2008). Expression of *GOI*s relative to *ACT2* (Arabidopsis) or *UBQ3* (soybean) reference genes (*REF*) was calculated according to Livak and Schmittgen (2001) with 2^(Ct *GOI*−Ct *REF*)^. Fungal mRNA (*PpTUB*) abundance in infected soybean was determined as described before (van de Mortel *et al*., 2007). For a list of used oligonucleotides, see Table S4.

### Scopoletin extraction

Soybean and Arabidopsis leaves were frozen in liquid nitrogen, ground, and extracted in 90% methanol (3 ml g^−1^ leaf powder), supplemented with 10 μm 4‐methylumbelliferone (Sigma‐Aldrich, https://www.sigmaaldrich.com) as the internal standard. Extraction was carried out on a rotating wheel for 16 h. Leaf debris was separated from the extract by centrifugation at full speed and for 15 min in a microfuge. The supernatant was subsequently evaporated in a Concentrator plus (Eppendorf, https://www.eppendorf.com). Pellets were dissolved in 150 μL methanol and subjected to HPLC.

### High‐performance liquid chromatography

20 μL of a methanol extract were separated by HPLC on a reversed‐phase C18 column (KROMAPLUS 100‐5‐C18 5.0; Prontosil, https://www.bischoff-chrom.de). The solvent gradient used for separation is shown in Table S5. A flow rate of 1 ml min^−1^ at a chromatography duration of 15 min was used for separation. Fluorescent components were detected at 335 nm excitation and 460 nm emission (FP 920 Intelligent Fluorescence Detector; Jasco). Scopoletin was identified by retention time of a scopoletin standard (Sigma‐Aldrich). Calibration curves with the standard were used to quantify the scopoletin concentration in leaf extracts.

### *In vitro* germination assays

*Pp* uredospores were suspended in water supplemented with 0.01% (v/v) Tween‐20 and different concentrations of a test compound at a density of 1 mg ml^−1^. All compounds except ascorbic acid (Sigma‐Aldrich) were dissolved as a stock solution in dimethyl sulphoxide (DMSO) and diluted to a final concentration of 0.1% DMSO. Ascorbic acid was dissolved in water. Approximately 500 μl suspension were sprayed on a glass slide covered with polyethylene foil to facilitate germination and appressoria formation. Subsequently, the glass slide was placed in a plastic container lined with soaked paper towels to ensure saturated humidity. After incubation for 16 h in the dark and at RT, germination rates were determined by bright‐field microscopy (Leica DMR, http://www.leica.com/). At least 100 spores were counted for each treatment.

### Determining the reversibility of *Pp* spore germination inhibition

For each treatment, two aliquots of a 1 mg ml^−1^ *Pp* uredospore suspension were supplemented with 0.01% (v/v) Tween‐20 and Orkestra (1:2000 dilution) or scopoletin (500 μm). Controls contained 0.01% (v/v) Tween‐20 but no Orkestra or scopoletin, respectively. One aliquot was sprayed onto polyethylene foil. After 16 h of incubation at high humidity, spore germination was counted as described above. Simultaneously the other aliquot was incubated for 16 h on a rotation wheel. Ungerminated spores were subsequently washed three times with water and spore germination rates determined 4 h after incubation on polyethylene foil at high humidity.

### Histochemical staining and quantification of intracellular ROS

For the detection of ROS in fungal pre‐infection structures, *Pp* uredospores were collected from SBR‐infected soybean leaves und suspended in 0.01% (v/v) Tween‐20 at a density of 1 mg ml^−1^. After germination on a rotating wheel at RT for 3 h the adequate test compound was added to a final concentration of 500 μm. Scopoletin and esculetin were pre‐solved as 500 mm stock solutions in DMSO and diluted 1:1000 to a final concentration of 0.1% DMSO. Ascorbic acid was pre‐solved as a 500 mm stock in H_2_O. 0.1% DMSO and H_2_O served as the controls. After incubation for 1 h on a rotating wheel at RT spores were stained with 2.5 μg ml^−1^ H_2_DCFDA for 30 min or treated with H_2_O (control). 20 μl spore suspension were gently distributed on glass slides, allowed to dry, and analyzed by confocal laser scanning microscopy using a TCS SP Spectral Confocal Microscope (Leica Microsystems, Wetzlar). Following uptake by the fungus, cleavage of the acetate groups by endogenous fungal esterases and oxidation, nonfluorescent H_2_DCFDA is converted to 2′,7′‐dichlorofluorescein (DCF) which was detected at 488 nm excitation and 500–550 nm emission. Quantification of the fluorescence signal was carried out by measuring the fluorescence intensity maximum in the region of interest across germinated hyphae using the Leica Application Suite X (LAS X, http://www.leica.com/) software.

Reactive oxygen species accumulation in *Pp* infection structures formed in Arabidopsis mutants with attenuated NHR was analyzed by subjecting leaves to DAB staining as described (Loehrer *et al*., 2008). In brief, inoculated leaves were harvested 2 dpi and incubated in DAB solution (1.68 mg ml^−1^, pH 3.8) for 8 h at high humidity. Only the leaf petiole was in contact with the solution. Therefore, uptake of the stain was driven by transpiration flow. To visualize DAB‐stained fungal and plant structures, leaves were fixed and cleared in saturated chloral hydrate (2.5 g ml^−1^), mounted in 50% (v/v) glycerol, and examined by bright‐field microscopy.

For Coomassie Brilliant Blue staining, *Pp* spores were incubated in 0.01% Tween‐20 for 3 h on a rotating wheel. After centrifugation for 1 min at 1000 ***g*** and RT, the supernatant was discarded and spores suspended in 0.6% (w/v) Coomassie Brilliant Blue (in ethanol). After 30 sec, spores were washed three times with ddH_2_O and analyzed by bright‐field microscopy.

Trypan blue staining was done by submerging inoculated leaves harvested 2 dpi in a nonphenolic trypan blue solution (10% (v/v) lactic acid, 10% (v/v) glycerol, 10% (v/v) H_2_O_2_, 70% (v/v) ethanol). Samples were heated to 80°C for 75 sec, cooled down to RT for 10 min and transferred to 2.5 g ml^−1^ chloral hydrate. Leaves were mounted in 50% (v/v) glycerol on glass slides and analyzed by bright‐field microscopy.

### ABTS assay

The antioxidant capacity of a compound was evaluated in an ABTS assay (Re *et al*., 1999). Stock solutions of the test compounds were prepared. Ascorbic acid and scopolin were dissolved in water, whereas umbelliferone, scopoletin and esculetin were dissolved in methanol. For ABTS^•+^ radical formation aqueous stock solutions of ABTS (7 mm) and potassium persulfate (2.4 mm) were mixed at a 1:1 ratio and incubated in the dark at RT for 16 h. The ABTS^•+^ solution was diluted with ethanol to an OD_734_ of 0.7 (± 0.02) and pre‐warmed to 37°C. 990 μl of the dilution were mixed with 10 μl of a test solution (final concentration 1–10 μm). Ethanol was used as a blank. After incubation for 7 min at 37°C, OD_734_ was measured and the absorbance inhibition determined relative to the blank control. Neither water nor methanol had any impact on the absorbance at 734 nm.

### Plant‐protection assays

Soybean and Arabidopsis plants were treated with 500 μm scopoletin in 0.1% (v/v) DMSO either before (−24 h, −6 h; pre‐application) or after (6 h, 24 h; post‐application) inoculation with *Pp*. Alternatively, spore suspensions were supplemented with scopoletin (co‐application). Control plants were inoculated with spore suspensions lacking scopoletin. Arabidopsis leaves were harvested 2 dpi and destained by incubation on chloral hydrate‐soaked paper towels (to prevent the removal of ungerminated spores). Fungal germination and penetration rates were determined in a Leica TCS fluorescence microscope with an epifluorescence filter (A‐513804, 340–380 nm extinction, 425 nm emission; Leica). At least 100 interaction sites were counted per leaf. Photos were taken with a digital JVC KYF camera (JVC, https://de.jvc.com). For quantifying rust severity on soybean, trifoliate leaves were scanned 10 dpi and the leaf area with rust symptoms determined using the image analysis software Assess 2.0 (Lamari, 2008).

### Analyzing scopoletin‐induced defence gene expression

Here, 5‐week‐old plants of the Arabidopsis *pen2* mutant were entirely sprayed with H_2_O, WP carrier (Syngenta, https://www.syngenta.de), 100 μm Benzo(1,2,3)thiadiazole‐7‐carbothioic acid *S‐*methyl ester (BTH) in WP carrier (Syngenta), 500 μm scopoletin TCI, (TCI Europe, https://www.tcichemicals.com), or 500 μm scopoletin in WP carrier. Control plants were left untreated. Three days after spraying half of the plants were challenged by syringe‐infiltration of leaves with tap water. The other half of plants was left untreated. Three hours after challenge, leaves of plants were harvested and the abundance of mRNA transcript of defence marker genes determined by RT‐qPCR.

## Conflict of interest

The authors HS, UC and CL are inventors of project‐linked patent WO/2016124515. HS is an employee of BASF Plant Science Company GmbH.

## Supporting information


**Figure S1.** MAPMAN illustration of secondary metabolism‐associated genes differentially expressed in Arabidopsis after inoculation with *Pp*.

**Figure S2.** *Pp*‐induced scopoletin accumulation in Arabidopsis *pen2* is not detectable by fluorescence microscopy.

**Figure S3.** Soybean *F6′H* transcripts and scopoletin are absent from healthy or *Pp*‐inoculated leaves of the SBR‐susceptible soybean cultivar W82.

**Figure S4.** In the incompatible interaction between *Pp* and soybean cultivar Ji wo dou neither Gm*F6′H* mRNA nor scopoletin accumulate in leaves.

**Figure S5.** ROS accumulation in *Pp* pre‐infection structures correlates with the density of the cytoplasm.

**Figure S6.** ROS accumulate in *Pp* infection structures after plant invasion.

**Figure S7.** Scopoletin does not interfere with alkalinization of the spore suspension.

**Table S1.** Expression levels and *Pp*‐induced activation or repression of selected PPP genes associated with scopoletin and flavonoid biosynthesis (depicted in Figure 1b).

**Table S2.** Expression levels and *Pp*‐induced activation or repression of genes from the MAPMAN pathway ‘secondary metabolism’ (depicted in Figure S1).

**Table S3.** Complete transcriptome data from wild‐type plants, and the *pen2* and *pen2 pad4 sag101* mutants 2 days after mock treatment or inoculation with *Pp*.

**Table S4.** Primer pairs used in this study.

**Table S5.** High‐performance liquid chromatography gradient used for scopoletin quantification.
