# Supplementary figures and images for: The Arabidopsis non‐host defence‐associated coumarin scopoletin protects soybean from Asian soybean rust
Source: Plant J. 2019 Jul 1;99(3):397–413. doi: 10.1111/tpj.14426 (PMC6852345; doi:10.1111/tpj.14426)

(a)

wild type

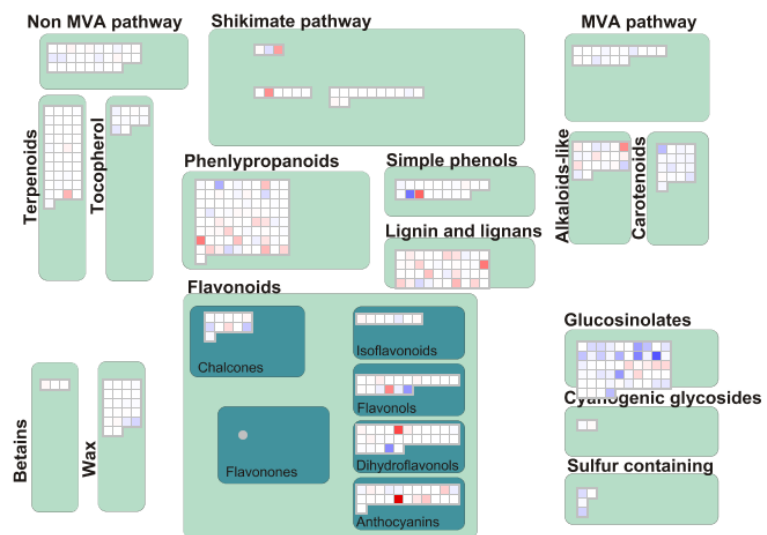

(b)

*pen2*

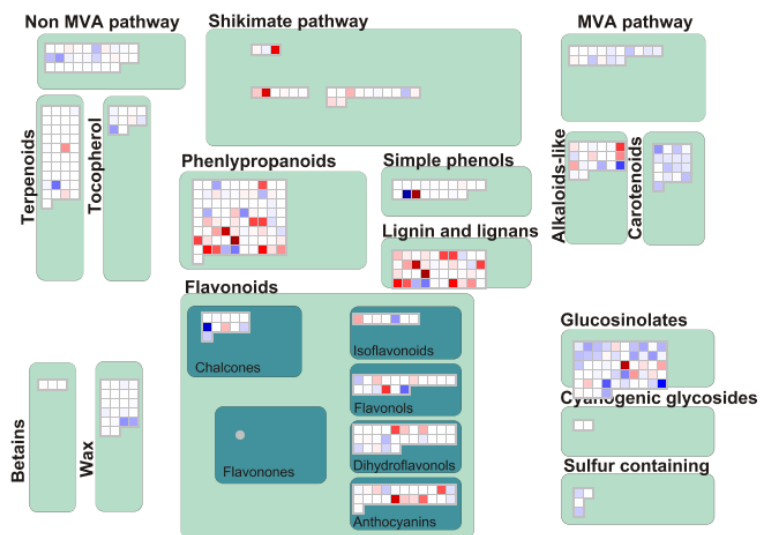

(c)

*pen2 pad4 sag101*

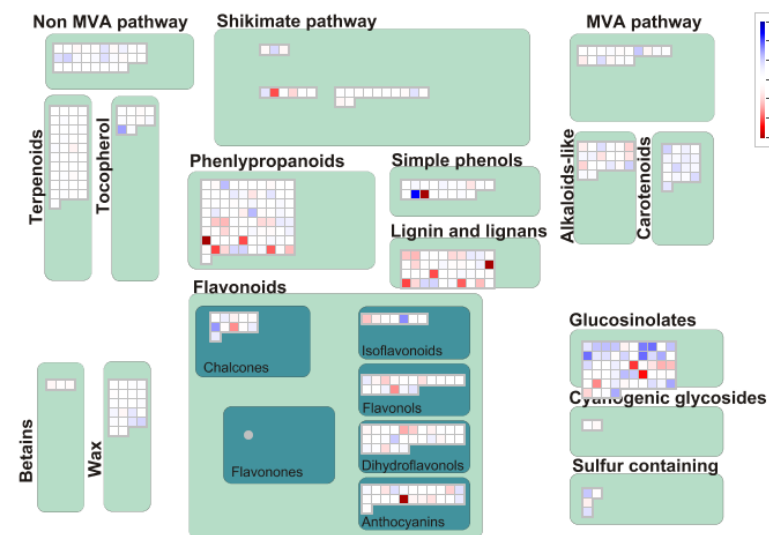

Supplement: Supplementary file 1 — Figure S1. MAPMAN illustration of secondary metabolism‐associated genes differentially expressed in Arabidopsis after inoculation with Pp. [file TPJ-99-397-s001.pdf]

Epifluorescence

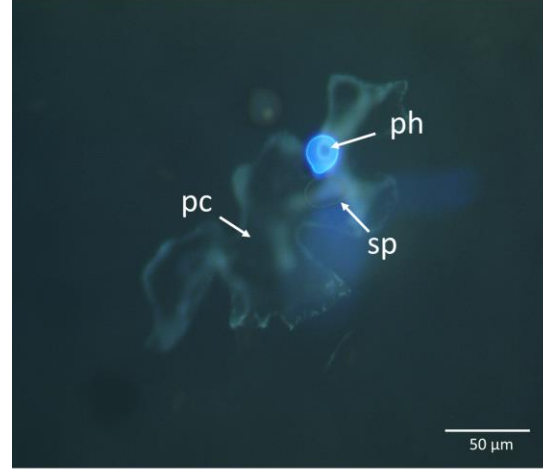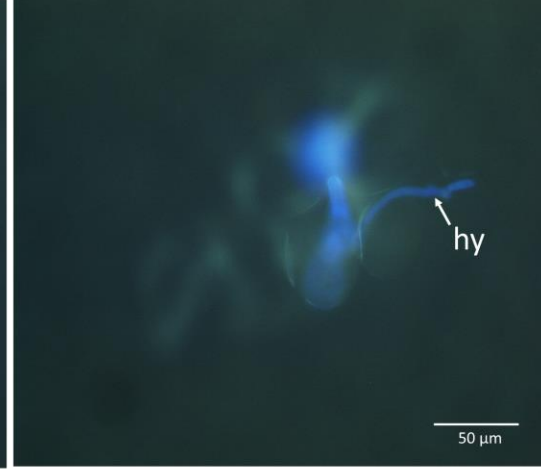

Brightfield

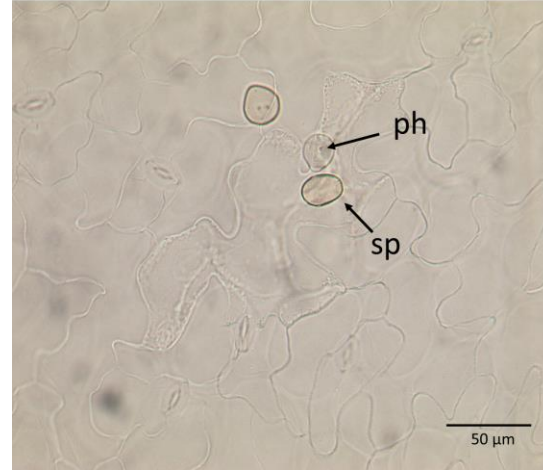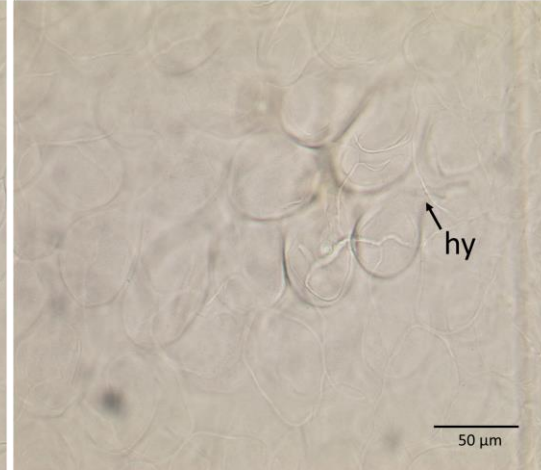

Supplement: Supplementary file 2 — Figure S2. Pp‐induced scopoletin accumulation in Arabidopsis pen2 is not detectable by fluorescence microscopy. [file TPJ-99-397-s002.pdf]

(a)

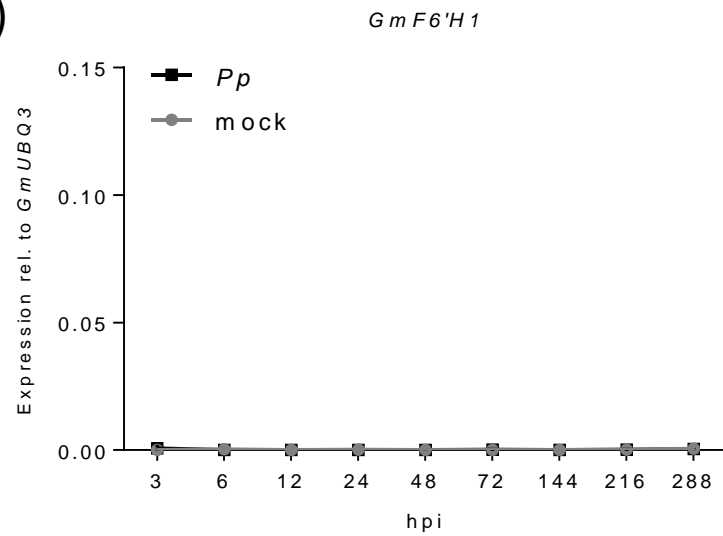

(b)

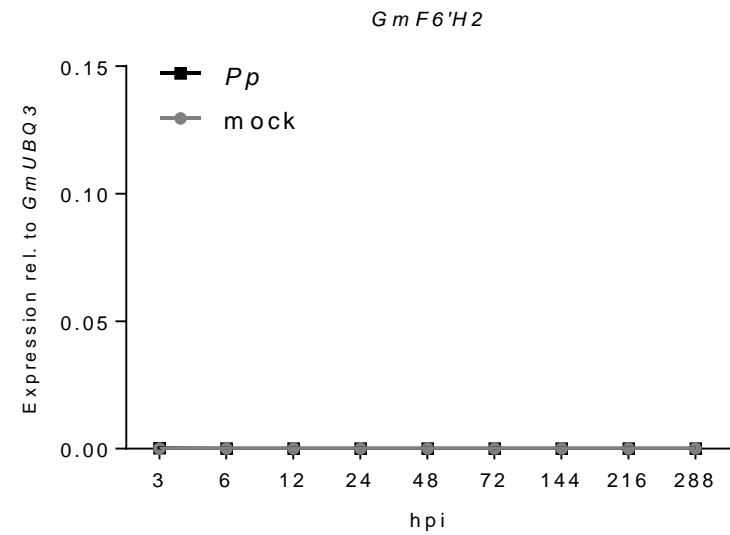

(c)

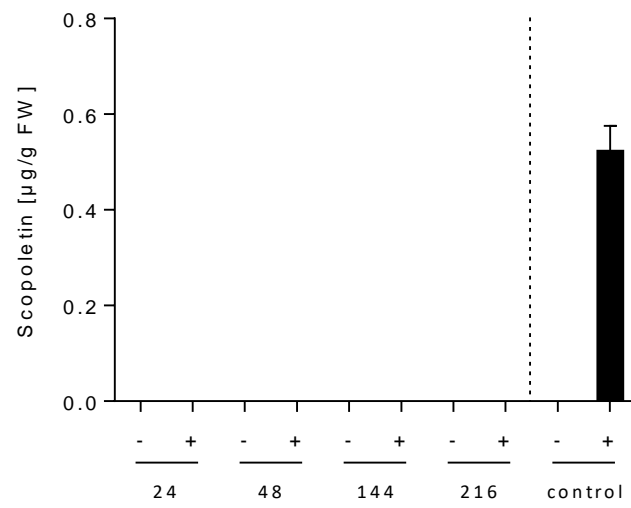

(d)

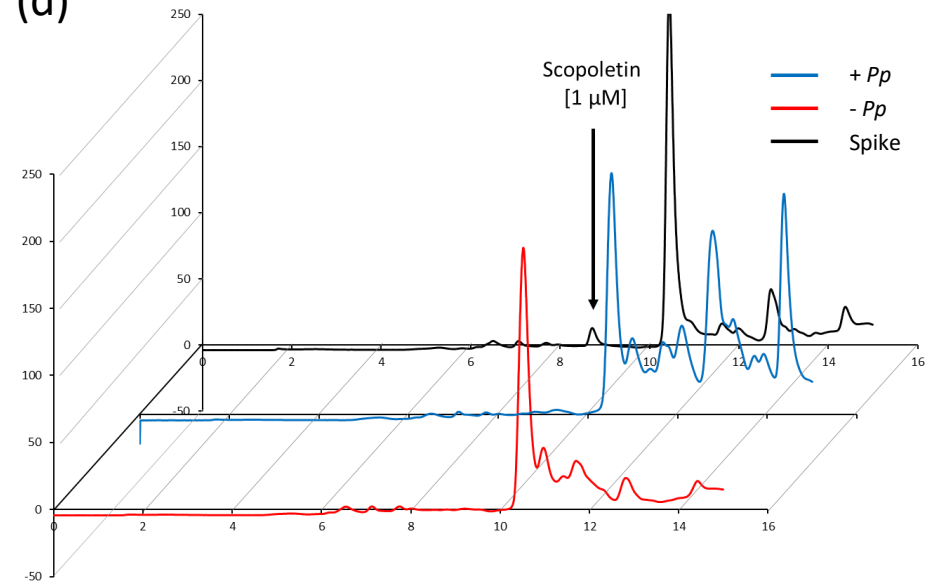

Supplement: Supplementary file 3 — Figure S3. Soybean F6′H transcripts and scopoletin are absent from healthy or Pp‐inoculated leaves of the SBR‐susceptible soybean cultivar W82. [file TPJ-99-397-s003.pdf]

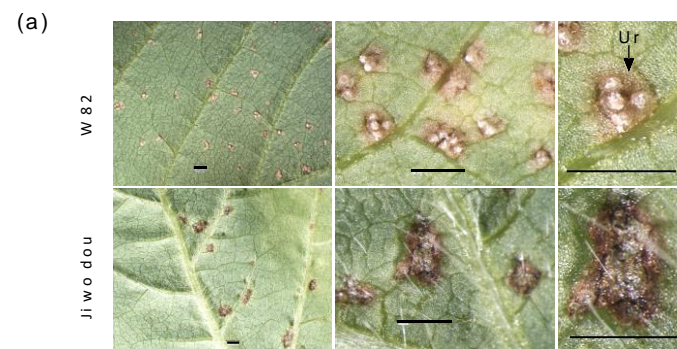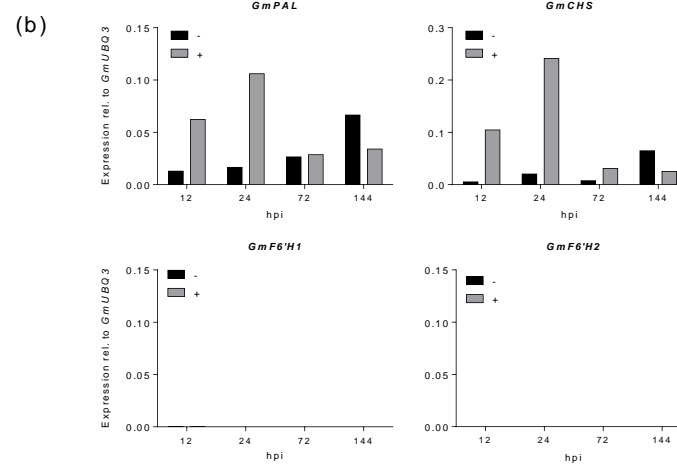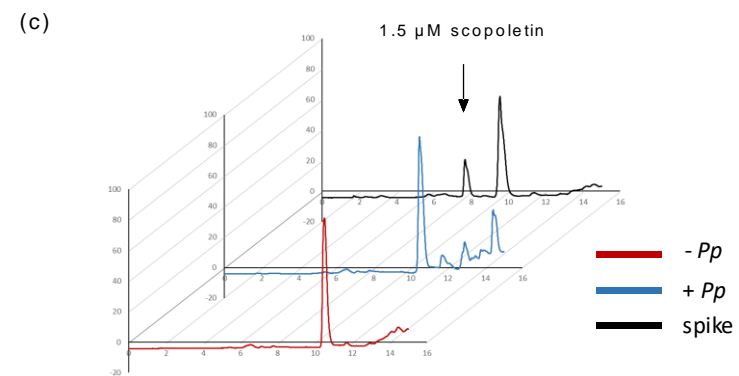

Supplement: Supplementary file 4 — Figure S4. In the incompatible interaction between Pp and soybean cultivar Ji wo dou neither GmF6′H mRNA nor scopoletin accumulate in leaves. [file TPJ-99-397-s004.pdf]

a)

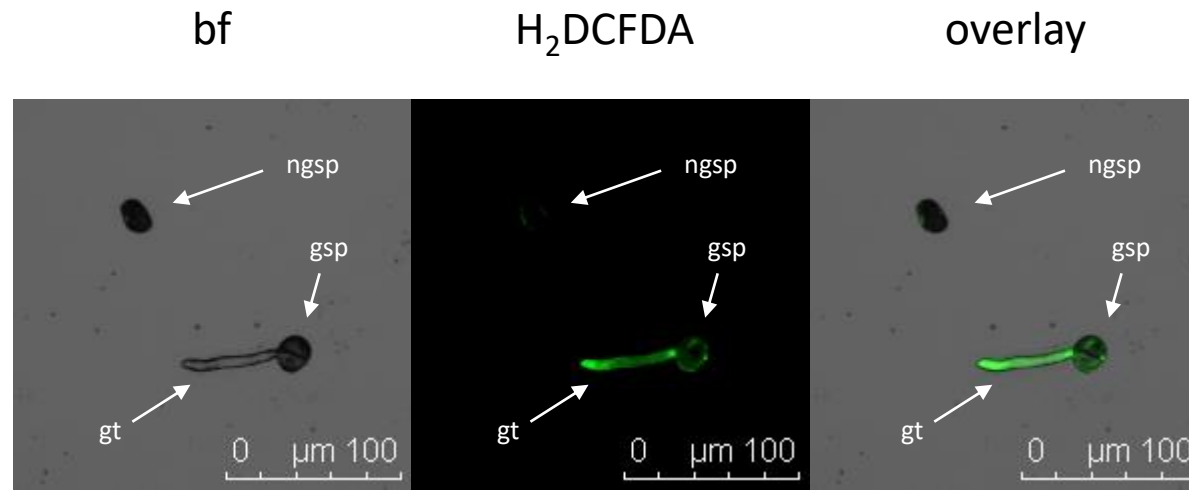

b)

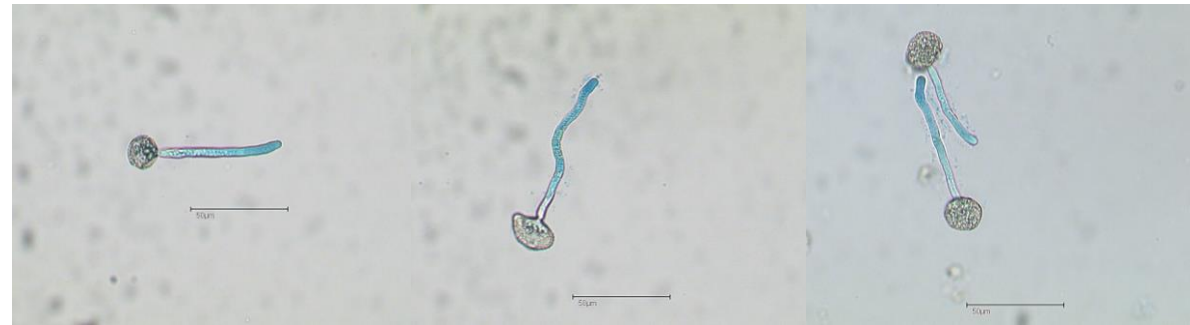

Supplement: Supplementary file 5 — Figure S5. ROS accumulation in Pp pre‐infection structures correlates with the density of the cytoplasm. [file TPJ-99-397-s005.pdf]

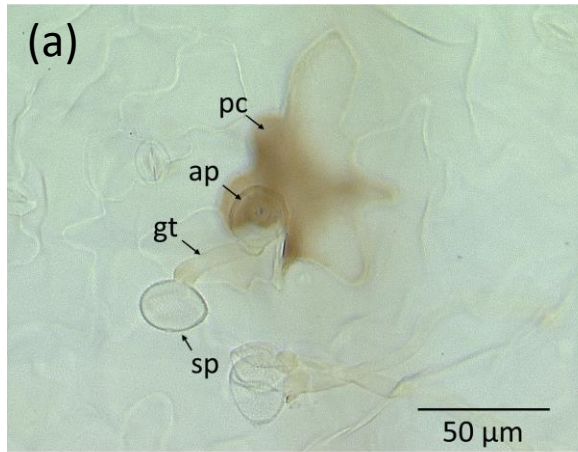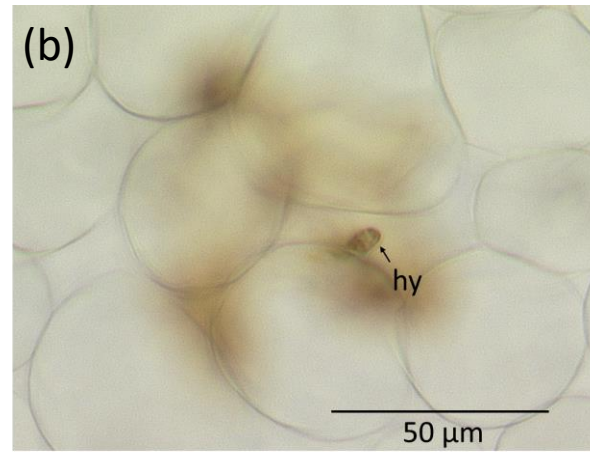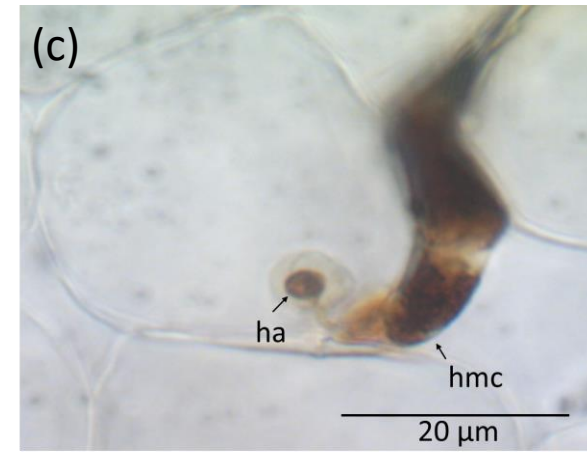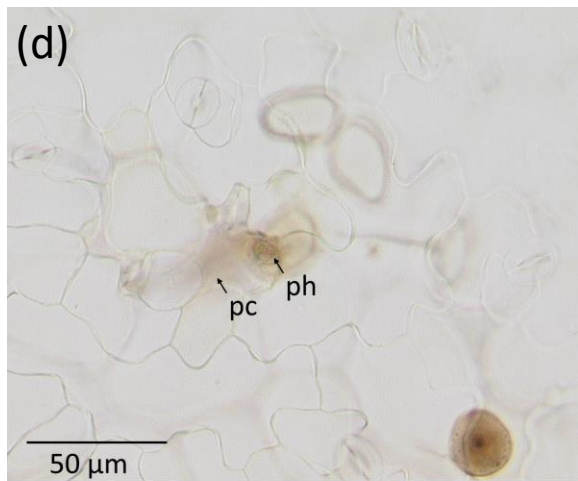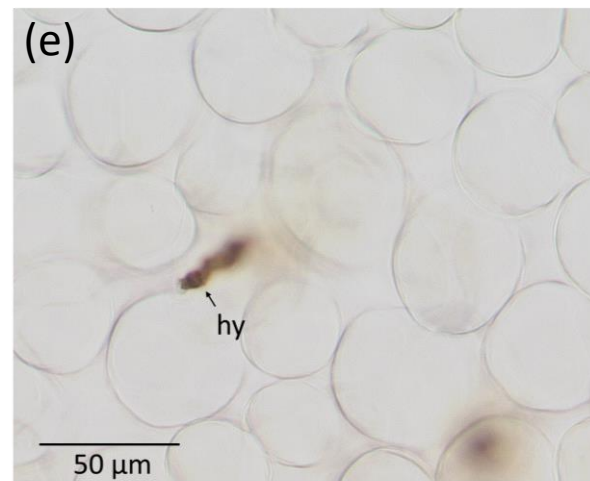

Supplement: Supplementary file 6 — Figure S6. ROS accumulate in Pp infection structures after plant invasion. [file TPJ-99-397-s006.pdf]

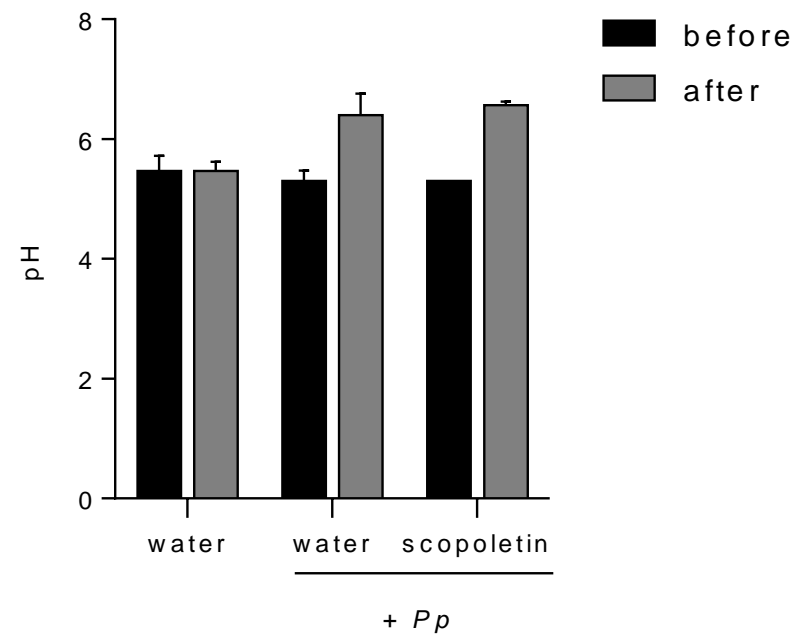

Supplement: Supplementary file 7 — Figure S7. Scopoletin does not interfere with alkalinization of the spore suspension. [file TPJ-99-397-s007.pdf]
